# The Patient Health Questionnaire-9 vs. the Hamilton Rating Scale for Depression in Assessing Major Depressive Disorder

**DOI:** 10.3389/fpsyt.2021.747139

**Published:** 2021-11-04

**Authors:** Simeng Ma, Jun Yang, Bingxiang Yang, Lijun Kang, Peilin Wang, Nan Zhang, Wei Wang, Xiaofen Zong, Ying Wang, Hanping Bai, Qingshan Guo, Lihua Yao, Li Fang, Zhongchun Liu

**Affiliations:** ^1^Department of Psychiatry, Renmin Hospital of Wuhan University, Wuhan, China; ^2^School of Information Engineering, Wuhan University of Technology, Wuhan, China; ^3^School of Health Sciences, Wuhan University, Wuhan, China; ^4^Department of Psychiatry, Jingmen No. 2 People's Hospital, Jingmen, China

**Keywords:** depression, PHQ-9, HAMD-17, assessment, item response theory

## Abstract

**Background:** The Hamilton Rating Scale for Depression (HAMD-17) has been used for several decades to assess the severity of depression. Multiple studies have documented defects in this scale and deemed it unsuitable for clinical evaluation. The HAMD-6, which is the abbreviated version of HAMD-17, has been shown to be effective in assessing the core symptoms of depression with greater sensitivity than HAMD-17. And the Patient Health Questionnaire-9 (PHQ-9) is suggested as an effective alternative to the HAMD-17 because of its simplicity and ease-of-use.

**Methods:** Research was completed involving 1,741 participants having major depressive disorder. Cronbach's alpha, intraclass correlation coefficient (ICC) and weighted Kappa analysis was used to determine the reliability of the scales. Pearson correlation analysis and factor analysis were used to analyze validity. Item response theory (IRT) was used to analyze psychological characteristics of items in both the HAMD-17 and PHQ-9.

**Results:** Reliability analysis showed that the Cronbach's alpha of the HAMD-17, HAMD-6 and PHQ-9 were 0.829, 0.764, and 0.893 respectively, and the ICC of the three scales ranged from 0.606 to 0.744. The Kappa score of the consistency of depression severity assessment was 0.248. Validity analysis showed that the PHQ-9 was a single factor structure, and the total score of the scale was strongly correlated with the HAMD-17 (*r* = 0.724, *P* < 0.001). The IRT analysis showed that the discrimination parameters of the PHQ-9 were higher than that of the HAMD-17 in all dimensions. The HAMD-6 had the lowest measurement accuracy in distinguishing the severity of depression, while the PHQ-9 had the highest measurement accuracy.

**Conclusion:** Results showed that the PHQ-9 was satisfactory in terms of reliability, validity and distinguishing the severity of depression. It is a simple, rapid, effective and reliable tool which can be used as an alternative to the HAMD-17 to assess the severity of depression.

## Introduction

Depression is a common psychiatric disorder with high morbidity and mortality and a leading contributor to the global burden of disease (1, 2). Currently, a diagnosis of depression is confirmed using through standardized interviews and assessment scales. The Hamilton Rating Scale for Depression (HAMD-17) is the most commonly used to estimate severity and response to treatment in patients who were already diagnosed with a depressive disorder (3–5). However, many questions have been raised about the effectiveness of HAMD-17 assessment and its inapplicability to clinical practice (6), mainly including the following:

The HAMD-17 is an observer-rated scale requiring clinician training in its use and takes 20–30 min to complete. The interview process depends entirely on the skill of the interviewer in eliciting the necessary information, and is not suitable for novices or inexperienced evaluators (7, 8).

The test-retest reliability of some items in this scale is poor, regarding loss of insight, genital symptoms, hypochondriasis and weight loss (6, 9, 10).

The HAMD-17 focuses not only on the core symptoms of depression, but also anxiety symptoms and side effects of drug treatment (11). Only six items correspond to symptoms used by experienced clinicians to formulate the overall assessment of depression severity (12, 13). These six items make up the abbreviated version of HAMD-17, known as HAMD-6, which has been shown to be effective in assessing the core (central) symptoms of depression with greater sensitivity than HAMD-17 (12–15). The HAMD-6 can measure acute episodes of antidepressant effects and is mainly used in the standardization of clinical practice and in antidepressant clinical trials (15).

Researchers have developed a new depression assessment scale, the Montgomery-Åsberg Depression Rating Scale (MADRS), which is superior to HAMD-17 in terms of internal reliability and sensitivity to change (16, 17). However, the MADRS requires professional training to use the same observer-rated scale in face-to-face patient interviews and remains time-consuming (8), which limits its wide application in outpatient and patient follow-up.

Compared with an observer-rated scale, a self-rating scale has some advantages. The self-rating scale requires patients to answer questions according to their own feelings, without a face-to-face evaluation by clinicians. It is more efficient and convenient, and can be widely used in outpatient settings, follow-up and epidemiological investigation.

The Patient Health Questionnaire-9 (PHQ-9) is a self-rating scale for screening and assessing depression which covering the DSM-IV algorithm for major depression (18). Studies have proven the effectiveness of the PHQ-9 in screening depression (19–24) and its ability to monitor the severity of depression (25, 26). Compared with HAMD-17, the PHQ-9 can reduce patient's treatment time and save medical costs, which is more suitable for clinical diagnosis and treatment. For example, it can quickly evaluate and track depressive symptoms in psychiatric outpatient and daily nursing follow-up, and clinicians can adjust treatment according to the results, so as to help patients achieve the best curative effect.

The purpose of this study is to explore whether the PHQ-9 can replace HAMD-17 to better assess the severity of depression. In addition, the PHQ-9 diagnostic algorithm and HAMD-6 scale were also included in the analysis. Three approaches are needed: analyze the reliability and validity of the PHQ-9 scale; use item response theory to analyze the ability of different items in the PHQ-9 and HAMD-17 scales to distinguish the severity of depression; and evaluate the test information function of the scales comparing the measurement precision and reliability of three scales.

## Methods

### Participants

This study was based on data from the Early Warning System and Comprehensive Intervention for Depression (ESCID) project collected from 15 hospitals in China from April 2019 to April 2021. All the clinicians who participated in the scale evaluation were trained in a consistent manner. Inclusion criteria of participants were: 18–55 years of age, having a junior high school education or higher, informed consent completed for participation and follow-up. In addition, all participants were diagnosed by an experienced psychiatrist and met the diagnostic criteria for major depressive disorder (MDD) recommended by the fifth edition of the Diagnostic and Statistical Manual of Mental Disorders. This diagnosis was made during a disease episode or remission.

### Measures

A researcher-designed socio-demographic questionnaire was used to obtain participant data including gender, age, educational level, occupation and relationship status. Depressive symptoms were assessed using three questionnaires: the PHQ-9, HAMD-6 and HAMD-17.

The PHQ-9 is a self-rating questionnaire which consists of nine depression criteria from the DSM-IV (18). The options for each item ranges from “none at all” (score 0) to “almost daily” (score 3), regarding how often each symptom has occurred in the patient during the previous 2 weeks (27). The total score ranges from 0 to 27, with the following results: no depression (0–4), mild depression (5–9), moderate depression (10–14), and severe depression (≥15) (18, 27). Commonly used screening depression methods include: (1) scoring threshold ≥10; (2) the diagnostic algorithm requires the score of 5 or more items ≥2, among which at least one item is depressive mood or anhedonia (28).

The HAMD-17 is one of the scales most commonly used by clinicians to evaluate depression symptoms (29–31). Most of the HAMD-17 items adopt the 5-level scoring method from 0 to 4 points, while a few items adopt the 3-level scoring method from 0 to 2 points. Each participant was evaluated by a professionally trained psychiatrist. Scores are categorized as 0–7 no depression, 8–16 mild depression, 17–23 moderate depression, and ≥24 severe depression. For the purpose of the study, depression was identified by a total score of 17.

The HAMD-6 was developed by Bech et al. which was a shorter version of the HAMD-17 scale, measures only depressed mood (item 1), guilt (Item 2), work and activities (Item 7), retardation (Item 8), anxiety psychic (Item 10), and general somatic symptoms (Item 13) (12). According to the DSM-IV criteria, these six selected HAMD-17 items represent the core symptoms of major depression.

In evaluating the concurrent validity of the PHQ-9 scale, three scales were completed by participants: Generalized Anxiety Disorder-7 (GAD-7) to evaluate anxiety symptoms (32), the Patient Health Questionnaire for Somatic Symptoms (PHQ-15) to evaluate somatic symptoms (33), and the Insomnia Severity Index (ISI) to evaluate insomnia (34).

### Statistics

#### Reliability and Validity

Cronbach's alpha was used to determine scale reliability and intraclass correction coefficient (ICC) was used for internal consistency. ICC < 0.40, poor internal consistency; ICC ranged from 0.40 to 0.75, good internal consistency; ICC >0.75, excellent internal consistency. Weighted Kappa analysis was used to assess the consistency between the PHQ-9 and HAMD-17 in assessing a participant's depression and its severity. Pearson correlation coefficient was used to evaluate the correlation between scores of each item and correlation among the scales. Factor analysis and correlation analysis were used to evaluate the validity of the scales. In addition, items of the PHQ-9 and HAMD-17 were divided into eight dimensions and matched with similar items on the two scales to analyze the correlation and compare item response theory (IRT) parameters between items in each dimension. Data were analyzed using SPSS version 22.0 (IBM Corp., Armonk, New York, United States), with the significance level set as α = 0.05, and statistical tests were two-tailed.

#### IRT Analysis

The IRT models should satisfy the basic assumptions of unidimensionality. A single-factor confirmatory factor analysis (CFA) model based on the raw categorical data with a weighted least squares means and variance-adjusted estimator (WLSMV estimation) was created using Mplus 8.3. The comparative fit index (CFI) value was >0.90, the Tucker-Lewis index (TLI) value was >0.90, and the root mean square error of approximation (RMSEA) value was < 0.10, supporting the unidimensionality assumption (35).

The item response data were composed of categorical data ordered according to the severity of symptoms, and the graded response model (GRM) conformed to the classification and ordered nature of the data. Each item in the model had two parameters: the discrimination parameter (a), which indicated the intensity of the relationship between the item and the potential severity, and the difficulty parameter (b), which indicated the severity of the symptom evaluated by the item (36–38).

Item characteristic function (ICF) is a mathematical model that describes the relationship among the ability level, item parameters and item response results, which is represented by charting an item characteristic curve (ICC). The horizontal axis represents the ability level of subjects and the vertical axis represents probability: the higher the ability value (θ), the greater the probability of the correct answer item.

An IRT uses the item information functions (IIFs) to express the certainty level of the information provided by the item or test when evaluating the trait state of the subject, which is equivalent to the reliability. The test information functions (TIFs) are the accumulation of information functions of items contained in a test. Higher information indicates greater precision for measuring a person's trait level. The standard error of measurement (SE) is the inverse function of the TIF. The SE was transformed into the reliability coefficient for different degrees of latent severity: *reliability (*θ*)* = *1 – SE (*θ*)*^2^. The R package “ltm” was used for the IRT analysis (39).

## Results

### Participant Characteristics and Scores of Scales

As shown in Table 1, the study population (*n* = 1,741) included 433 males (24.9%) and 1,308 females (75.1%). Most of the depressed patients were 18–26 years of age (83.9%), unmarried (88.7%), college students (70.5%), and had an undergraduate education (80.8%). The mean (*SD*) scores for the PHQ-9, HAMD-17, HAMD-6, GAD-7, PHQ-15 and ISI were 15.4(6.7), 18.2(7.7), 8.8(3.6), 10.2(5.8), 12.3(6.0), and 11.3(6.6) respectively.

**Table 1:** Socio-demographic characteristics of participants (*n* = 1,741).

**Variables**	**Frequency/Mean**	**Percentage (%) / SD**
**Gender**		
Male	433	24.9
Female	1,308	75.1
**Relationship status**		
Single	1,145	65.8
Has a partner	400	23
Married^a^	196	11.3
**Occupation**		
College student	1,227	70.5
Professional^b^	514	29.5
**Education level**		
High school or less	152	8.7
Undergraduate	1,407	80.8
Postgraduate or higher	182	10.5
**Age (years)**		
18–22	1,155	66.3
23–26	306	17.6
27–30	128	7.4
>30	152	8.7
PHQ-9	15.4	6.7
HAMD-6	8.8	3.6
HAMD-17	18.2	7.7
GAD-7	10.2	5.8
PHQ-15	12.3	6.0
ISI	11.3	6.6

a *The married category included widowed and divorced participants*.

b *People who have prior work history*.

*PHQ-9, the Patient Health Questionnaire-9; HAMD, the Hamilton Rating Scale for Depression; GAD-7, the Generalized Anxiety Disorder-7; PHQ-15, the Patient Health Questionnaire for Somatic Symptoms; ISI, the Insomnia Severity Index*.

### Reliability

As shown in Supplementary Table 1, Cronbach's alpha for the HAMD-17 and HAMD-6 was 0.829 and 0.764, and it was 0.893 for the PHQ-9. The ICC between the PHQ-9 scores, HAMD-17 scores and HAMD-6 scores ranged from 0.606 to 0.744, demonstrating good internal consistency (Supplementary Table 2).

As shown in Supplementary Table 3, based on the cut-off points, consistency analysis between the depression severity obtained by PHQ-9 and HAMD-17 revealed the Kappa coefficient of 0.248 (95% CI, 0.219–0.277, *P* < 0.001). Based on depression / no depression, the Kappa coefficient was 0.476 (95% CI, 0.435–0.517, *P* < 0.001). According to PHQ-9 diagnostic algorithm to distinguish depression / no depression, the Kappa coefficient of HAMD-17 and PHQ-9 was 0.526 (95% CI, 0.505–0.547, *P* < 0.001), indicating a moderate level of consistency.

### Validity

#### Concurrent Validity

As shown in Table 2, Pearson's correlation analysis of PHQ-9 with GAD-7, PHQ-15, and ISI was used to evaluate content validity, and the correlation coefficients were 0.736, 0.682, and 0.605 (*P* < 0.001), indicating strong correlation. Total scores of the PHQ-9 and HAMD-17 also had a strong correlation (*r* = 0.724, *P* < 0.001). However, compared with the total scores of PHQ-9, the correlation between PHQ-9 diagnostic algorithm and other scales decreased, ranging from 0.475 to 0.585. HAMD-6 showed a strong correlation with all scales except ISI scale, ranging from 0.484 to 0.914. The correlation analysis of matching items is shown in Supplementary Table 4. There is a significant correlation between matching items, and the correlation coefficients ranged from 0.257 to 0.678. The suicide dimension had strong correlation (*r* = 0.678, *P* < 0.001) and only the attention and anxiety dimension showed weak correlation (*r* = 0.257–0.338, *P* < 0.001). In addition, the results showed that there was no correlation between “insight (H17)” and all items of the PHQ-9.

**Table 2:** Pearson's correlation analysis.

**Scale**	**Diagnostic algorithm of PHQ-9**	**PHQ-9**	**HAMD-17**	**HAMD-6**	**GAD-7**	**PHQ-15**	**ISI**
Diagnostic algorithm of PHQ-9	1						
PHQ-9	0.807**	1					
HAMD-17	0.585**	0.751**	1				
HAMD-6	0.572**	0.724**	0.914**				
GAD-7	0.569**	0.736**	0.648**	0.606**	1		
PHQ-15	0.498**	0.682**	0.635**	0.608**	0.605**	1	
ISI	0.475**	0.605**	0.600**	0.484**	0.554**	0.612**	1

** *Correlation is significant at the 0.01 level (2-tailed)*.

*PHQ-9, the Patient Health Questionnaire-9; Diagnostic algorithm of PHQ-9, the score of 5 or more items ≥2, among which at least one item is depressive mood or anhedonia; HAMD, the Hamilton Rating Scale for Depression; GAD-7, the Generalized Anxiety Disorder-7; PHQ-15, the Patient Health Questionnaire for Somatic Symptoms; ISI, the Insomnia Severity Index*.

#### Construct Validity

The KMO value was 0.92, indicating adequacy for factor analysis, and Bartlett's sphericity value was also statistically significant (χ^2^ = 7564.43, *P* < 0.001). On principal component analysis, only the eigenvalues of the first principal components were >1, which explained the total data variation of 54.68%. The results showed that all items in the PHQ-9 exhibited the same characteristics.

### IRT Analysis

#### Dimensionality

Previously, exploratory factor analysis has been used to prove that PHQ-9 is a single-factor structure. As shown in Supplementary Table 1, the one-factor CFA model for the HAMD-17 fit well to the validation sample data (CFI = 0.93, TLI = 0.92, RMSEA = 0.065). Likewise, similar results were obtained in HAMD-6 data (CFI = 0.98, TLI = 0.96, RMSEA = 0.080). The one-factor CFA model for the PHQ-9 scale fit adequately to the validation sample data. Although the RMSEA was >0.10, both the CFI and TLI were within the acceptable ranges (CFI = 0.97, TLI = 0.97, RMSEA = 0.107). Overall, these fit indices suggested that the total items reflect sufficient unidimensionality for the purposes of calibrating the two scales simultaneously.

#### Discrimination Values

The difficulty and discrimination values for all items in the two scales are displayed in Table 3. For the PHQ-9 items, the discrimination values ranged from 1.45 to 2.80; “feeling down, depressed, or hopeless (P2)” consistently showed the highest level of discrimination, while “sleep disturbance (P3)” consistently showed the lowest level of discrimination. For the HAMD-17 items, the discrimination values ranged from −0.02 to 1.73. The two items with the highest discrimination were “depressed mood (H1)” and “suicide (H3),” while “insight (H17),” “hypochondriasis (H15),” and “genital symptoms (H14)” showed the lowest level of discrimination.

**Table 3:** Item content and IRT item parameter estimates.

**Symptom**	**Items on the PHQ-9**	**a(SE)**	**b1(SE)**	**b2(SE)**	**b3(SE)**	**Items on the HAMD-17**	**a(SE)**	**b1(SE)**	**b2(SE)**	**b3(SE)**	**b4(SE)**
Interest	P1: Little interest or pleasure in doing things	2.41(0.10)	−1.88(0.07)	−0.55(0.88)	0.29(0.06)	**H7: Work and activities**	1.42(0.07)	−1.87(0.09)	−0.62(0.07)	1.74(0.24)	3.00(3.79)
Mood	P2: Feeling down, depressed, or hopeless	2.80(0.11)	−1.75(0.06)	−0.42(0.07)	0.46(0.07)	**H1: Depressed mood (sadness, hopeless, helpless, worthless)**	1.73(0.07)	−1.87(0.08)	−0.75(0.08)	0.34(0.06)	2.27(1.13)
Sleep	P3: Trouble falling or staying asleep, or sleeping too much	1.45(0.07)	−1.92(0.09)	−0.52(0.07)	0.47(0.07)	H4: Insomnia: early in the night;	1.07(0.06)	−0.55(0.06)	0.64(0.08)		
						H5: Insomnia: middle of the night;	0.80(0.06)	−1.05(0.10)	1.61(0.16)		
						H6: Insomnia: early hours of the morning	0.78(0.06)	−0.24(0.07)	1.73(0.21)		
Energy	P4: Feeling tired or having little energy	2.46(0.10)	−1.99(0.07)	−0.71(0.09)	0.22(0.07)	**H13: General somatic symptoms**	1.41(0.07)	−1.10(0.07)	0.61(0.05)		
Diet	P5: Poor appetite or overeating	1.49(0.07)	−1.37(0.07)	−0.14(0.05)	0.96(0.11)	H12: Somatic symptoms gastro-intestinal	1.23(0.07)	−0.30(0.05)	2.03(0.32)		
Pessimism	P6: Feeling bad about yourself	2.38(0.10)	−1.42(0.06)	−0.33(0.05)	0.48(0.06)	**H2: Feelings of guilt**	1.35(0.07)	−1.19(0.07)	−0.05(0.04)	2.01(0.38)	4.73(48.19)
Attention and anxiety	P7: Trouble concentrating on things	1.63(0.07)	−1.43(0.07)	−0.18(0.05)	0.84(0.10)	**H8: Retardation**	1.06(0.06)	−1.52(0.10)	1.88(0.19)	6.18(71.01)	/^a^
	P8: Moving or speaking so slowly that other people could have noticed− Or so fidgety or restless that you have been moving a lot more than usual	1.55(0.08)	−0.45(0.05)	0.59(0.06)	1.68(0.41)	H9: Agitation	0.98(0.06)	−1.04(0.08)	1.20(0.11)	2.99(0.98)	3.40(4.66)
						**H10: Anxiety psychic**	1.15(0.06)	−1.81(0.10)	−0.12(0.05)	1.27(0.15)	5.54(45.81)
Suicide	P9: Thoughts that you would be better off dead, or thoughts of hurting yourself in some way	2.04(0.09)	−0.43(0.04)	0.78(0.08)	1.64(0.75)	H3: Suicide	1.72(0.08)	−0.54(0.05)	0.17(0.04)	0.73(0.11)	2.79(3.46)
Other						H11: Anxiety somatic	1.27(0.06)	−1.38(0.08)	0.09(0.04)	1.81(0.29)	4.21(13.31)
						H14: Genital symptoms	0.54(0.07)	1.54(0.20)	/^b^		
						H15: Hypochondriasis	0.52(0.05)	0.39(0.10)	2.37(0.38)	8.73(11.64)	14.42(1056.55)
						H16: Loss of weight	0.75(0.07)	1.32(0.12)	2.47(0.56)		
						H17: Insight	−0.02(0.07)	−97.38(384.19)	−227.05(915.78)		

a *Stupor was excluded in this study because the patient was unable to cooperate with the assessment*.

b *This option indicates that it is not certain, or that it is not suitable for the patient (not included in the total score)*.

*PHQ-9, the Patient Health Questionnaire-9; HAMD, the Hamilton Rating Scale for Depression*.

*Bold text represented HAMD-6 items*.

By matching and comparing the items of the two scales, it was found that the discrimination values of PHQ-9 items in each dimension were greater than that of HAMD-17, indicating that PHQ-9 items could better distinguish patients with differences in the severity of their depression.

#### Category Characteristics Curves

**Figure 1:**
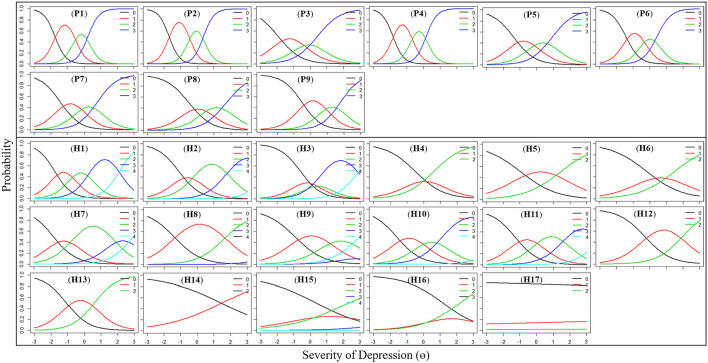
Category characteristics curves for the PHQ-9 and HAMD-17 item.

The category characteristics curves for the items are shown in Figure 1. The horizontal axis represents the depression level of the subjects and the vertical axis represents the probability. It shows that the probability of responding to each category correlates with the underlying level of depression. For example, for the P2 item, patients with a latent trait value θ >-1.68 were most likely to choose “0”; those with θ between −1.68 and −0.44 were most likely to choose “1,” those with θ between −0.44 and 0.35 were most likely to choose “2,” while those with θ between ≥0.35 were most likely to choose “3.” The greater the severity of the depression, the greater the probability of choosing a higher score. All 9 items on the PHQ-9 scale performed very well. However, some items of the HAMD-17 scale did not conform to this rule, including items H1, H3, H4, H8, H9, H15, H16, and H17. In particular, the responses of “insight (H17)” to these categories did not seem to correlate with the subject's underlying level of depression.

#### Test Information Functions

**Figure 2:**
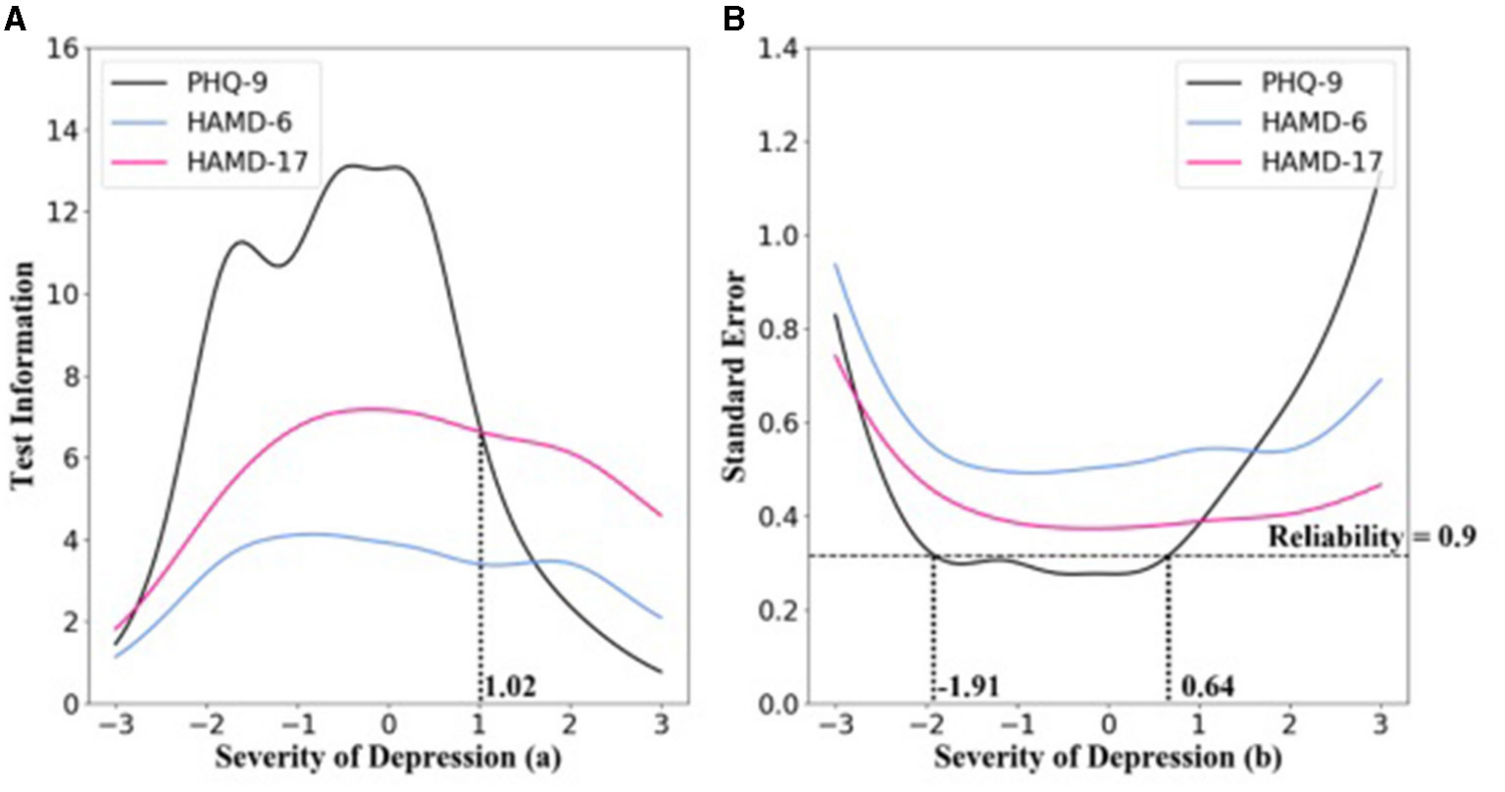
Test information functions (TIFs) curves of PHQ-9, HAMD-6, and HAMD-17. **(A)** Test information function for PHQ-9, HAMD-6, and HAMD-17. **(B)** Standard error for PHQ-9, HAMD-6, and HAMD-17. Below the dotted line indicates that the reliability was >0.9.

Figure 2 displays the test information functions (TIFs) curves and associated standard errors of the measurements for the PHQ-9, HAMD-6, and HAMD-17. The information was distributed near the average of the latent trait. The peak information value of PHQ-9 was at θ = −0.36 (information value = 13.11, *SE* = 0.38). The range of the highest measurement accuracy was θ from −1.91 to 0.64, where the information value was >10.01, the standard error was < 0.32, and the corresponding reliability was >0.9. The peak information value of HAMD-17 was at θ = −0.20 (information value = 7.17, *SE* = 0.37), and the corresponding reliability was 0.86. The peak information value of HAMD-6 was at θ = −0.82 (information value = 4.13, *SE* = 0.49), and the corresponding reliability was 0.76. The HAMD-6 had the lowest measurement accuracy in distinguishing the severity of depression.

As evident in Figure 2, compared with HAMD-17, the PHQ-9 provided increased information regarding most of the subjects, and the measurement precision of the scale was satisfactory. However, when θ was >1.02, HAMD-17 provided more information, and the corresponding HAMD-17 score was >49.

## Discussion

In this study, it was verified that the PHQ-9 has acceptable reliability and validity. By analyzing the discrimination parameters of matching items, it was found that each dimension of the PHQ-9 is better than HAMD-17 in distinguishing the severity of depression. These results support the use of the PHQ-9 as an alternative to the HAMD-17 for assessing the severity of depression.

The current study confirmed that the PHQ-9 and HAMD-17 have high reliability with good internal consistency, and there is general or moderate correlation between the disease severity as assessed by the PHQ-9 and HAMD-17 (40). Clinical studies have demonstrated a moderate degree of consistency between self-rating and observer rating (41). Approximately one-third of patients have been found to have inconsistent scores (42, 43). This situation may be related to the inconsistent items of the scale, clinician assessment bias (44), education level (45), gender (45), and high neuroticism (46).

Consistent with previous studies, the factor structure of the PHQ-9 supports the notion that all items of the PHQ-9 are measuring the same affective factors (47–49). There was a strong correlation between PHQ-9 total scores and GAD-7, PHQ-15, ISI total scores, supporting the validity of PHQ-9 for assessing depression severity.

The total score was used to evaluate the severity of depression, and the weight of different symptoms was ignored. This study identified items with different psychometric characteristics, including different levels of difficulty and discrimination. “Feeling down, depressed, or hopeless (P2)” consistently showed the highest level of discrimination, while “depressed mood (H1)” consistently showed the highest level of discrimination. Emotional symptoms are more conducive to distinguishing patients with depression, which is consistent with the diagnostic criteria of MDD. Items with high levels of discrimination provided more information. High discriminative items help clinicians identify MDD more effectively.

The discrimination parameters of each item in the PHQ-9 were above 1.45, indicating that each item contributed significantly to the test information. The category characteristics curves of the PHQ-9 show that with an increase of depression severity θ, patients are more likely to choose a higher level of option on each item. The eight dimensions were obtained by matching the item of the PHQ-9 and HAMD-17, and the item discrimination parameter of each dimension of the PHQ-9 was larger than that of HAMD-17, indicating that the PHQ-9 can better distinguish the severity of depression.

The category characteristics curve of “insight (H17)” almost did not correlate with the severity of depression. Previous studies have also reported that the reliability of “insight (H17)” is poor (30). Insight includes three different dimensions, namely, understanding of mental illness, compliance with treatment and attribution of symptoms to disorder (50). Much of the previous research on insight about mental illness has focused on schizophrenia or bipolar disorder (51–53). There was a positive correlation between the degree of insight and depressive symptoms in schizophrenic patients (51, 54). In patients with depression, higher CES-D scores were significantly associated with intact insight for awareness of illness (55). However, the correlation between “insight (H17)” and depression score was very weak in this study, which provided inadequate information for identifying the severity of depression. The tendency of patients to choose option “0–2” did not change with the severity of depression. The authors speculate that this may be related to the following factors: inadequate explanation by clinicians; insufficient understanding of the item even though most of the patients had bachelor's degree or above; and inappropriate translation of the item into Mandarin. These reasons lead to the poor discrimination of the “insight” item of the HAMD-17 in this study.

In addition, “genital symptoms (H14)” also provided poor discrimination. The fact that the patients were mainly young unmarried college students, their sexual interest decline is not enough to affect daily life, and patients may ignore this symptom, could explain the poor differentiation of this item. And the reluctance of most Chinese people to talk about sex in public also affected the results which is related to national culture. These items with poor discrimination may affect the HAMD-17 assessment score, thus affecting the accuracy of the assessment. Continued use of items with low discrimination will underestimate the strength of actual treatment effects.

For some items on the HAMD-17, the likelihood of receiving a rating of “4” was very low even when overall depression was severe, such as “feelings of guilt (H2),” “retardation (H8),” “anxiety psychic (H10),” “anxiety somatic (H11),” and “hypochondriasis (H15).” This is consistent with previous research findings (56). For many items on the HAMD-17, the rating scheme is not ideal, which reduces the ability of the HAMD-17 to detect changes.

Although the measurement precision of the PHQ-9 was lower than that of the HAMD-17, when θ was >1.02, the corresponding HAMD-17 score was 49. This type of patient is rarely encountered in a clinic setting, that is to say, the PHQ-9 can meet clinical needs and provide accurate assessment for most patients.

Previous studies have shown that HAMD-6 is superior to HAMD-17 in determining core symptoms and changes with treatment. In this study, HAMD-6 was not as accurate as HAMD-17 and PHQ-9 in assessing depression severity. The possible reason is that HAMD-6 lacked of the major DSM- IV criterion in diagnosis of major depression and was originally designed to be used in clinical trials to detect changes in core symptoms (15).

The results of our study can be extended to clinical practice, that is, PHQ-9 can be preferred, HAMD-17 and HAMD-6 are not recommended if only the severity of patients with depression needs to be assessed, such as in outpatient service, daily follow-up care and epidemiological investigation. Because compared with these two scales, PHQ-9 is more time-saving, convenient and accurate.

This study has several limitations. First, patients in complete stupor or having hypochondriacal delusions were excluded from the study. It is necessary to expand the sample size to confirm the consistency of the results. Second, although clinicians may have completed the consistency training, evaluation findings may still be different. Future studies need to explore the discrepancies in evaluation and the psychological characteristics of different items of the HAMD-17 under more strict supervision. Third, the research data were cross-sectional and did not study the sensitivity of the PHQ-9 and HAMD-17 to treatment, which needs to be verified by incorporating appropriate research designs in future studies.

## Conclusion

It is time to take seriously the clinical measurement limitations of HAMD-17 and explore a new “gold standard.” The current study showed that the PHQ-9 was satisfactory in terms of reliability, validity and distinguishing the severity of depression. The PHQ-9 is a simple, rapid, effective and reliable tool, which can be used as an alternative to the HAMD-17 to assess the severity of depression.

## Data Availability Statement

The raw data supporting the conclusions of this article will be made available by the authors, without undue reservation.

## Ethics Statement

The studies involving human participants were reviewed and approved by the Ethics Committee of Renmin Hospital of Wuhan University. The patients/participants provided their written informed consent to participate in this study.

## Author Contributions

SM, JY, LY, LF, and ZL: drafted the manuscript. SM and JY: contributed to data analysis, results, and finalized the manuscript. SM, JY, BY, LK, PW, NZ, WW, XZ, YW, HB, QG, LY, LF, and ZL: make important contributions to data collection. All authors have read and approved the final manuscript.

## Funding

This work was supported by grants from the National Key R&D Program of China (Grant No. 2018YFC1314600) and the National Natural Science Foundation of China (Grant No. 81771472).

## Conflict of Interest

The authors declare that the research was conducted in the absence of any commercial or financial relationships that could be construed as a potential conflict of interest.

## Publisher's Note

All claims expressed in this article are solely those of the authors and do not necessarily represent those of their affiliated organizations, or those of the publisher, the editors and the reviewers. Any product that may be evaluated in this article, or claim that may be made by its manufacturer, is not guaranteed or endorsed by the publisher.
