# Supplementary material for: The Patient Health Questionnaire-9 vs. the Hamilton Rating Scale for Depression in Assessing Major Depressive Disorder
Source: Front Psychiatry. 2021 Nov 4;12:747139. doi: 10.3389/fpsyt.2021.747139 (PMC8599822; doi:10.3389/fpsyt.2021.747139)
Supplement: Supplementary file 1 [file Table_1.DOC]

Supplementary Table 1. Reliability and confirmatory factor analysis

| Scale | Cronbach's Alpha | Confirmatory Factor Analysis (CFA) | | |
| --- | --- | --- | --- | --- |
|  |  | CFI | TLI | RMSEA |
| HAMD-17 | 0.829 | 0.93 | 0.92 | 0.065 |
| HAMD-6 | 0.764 | 0.98 | 0.96 | 0.080 |
| PHQ-9 | 0.893 | 0.97 | 0.97 | 0.107 |

Abbreviations: PHQ-9, the Patient Health Questionnaire-9; HAMD, the Hamilton Rating Scale for Depression.

Supplementary Table 2. Intraclass correlation coefficient analysis between the three scales

| Scale | ICC value | 95%CI | P-value |
| --- | --- | --- | --- |
| HAMD-17 and HAMD-6 | 0.702 | 0.678-0.725 | <0.001 |
| HAMD-17 and PHQ-9 | 0.744 | 0.722-0.764 | <0.001 |
| HAMD-6 and PHQ-9 | 0.606 | 0.575-0.635 | <0.001 |

Abbreviations: PHQ-9, the Patient Health Questionnaire-9; HAMD, the Hamilton Rating Scale for Depression.

Supplementary Table 3. Weighted Kappa analysis of PHQ-9 and HAMD-17

| Variables | Classification | HAMD-17 | PHQ-9 | Kappa coefficient | 95% CI | P-value |
| --- | --- | --- | --- | --- | --- | --- |
| Depression severity | No | 0-7 | 0-4 | 0.248 | 0.219-0.277 | <0.001 |
|  | Mild | 8-16 | 5-9 |  |  |  |
|  | Moderate | 17-23 | 10-14 |  |  |  |
|  | Severe | ≥24 | ≥15 |  |  |  |
| Depression / no depression | No | 0-16 | 0-9 | 0.476 | 0.435-0.517 | <0.001 |
|  | Yes | ≥17 | ≥10 |  |  |  |
| Depression / no depression | No | 0-16 | Diagnostic algorithm | 0.526 | 0.505-0.547 | <0.001 |
|  | Yes | ≥17 |  |  |  |  |

Abbreviations: PHQ-9, the Patient Health Questionnaire-9; Diagnostic algorithm: the score of 5 or more items ≥2, among which at least one item is depressive mood or anhedonia; HAMD, the Hamilton Rating Scale for Depression.

Supplementary Table 4. Pearson’s correlation analysis of PHQ-9 and HAMD-17 items

|  | P1 | P2 | P3 | P4 | P5 | P6 | P7 | P8 | P9 |
| --- | --- | --- | --- | --- | --- | --- | --- | --- | --- |
| H1 | .475** | .584** | .325** | .472** | .359** | .502** | .374** | .341** | .436** |
| H2 | .336** | .410** | .254** | .334** | .263** | .531** | .343** | .334** | .401** |
| H3 | .408** | .486** | .302** | .401** | .292** | .502** | .324** | .287** | .678** |
| H4 | .299** | .314** | .526** | .335** | .289** | .279** | .273** | .242** | .275** |
| H5 | .234** | .241** | .378** | .221** | .247** | .229** | .219** | .186** | .215** |
| H6 | .230** | .257** | .260** | .209** | .227** | .224** | .209** | .189** | .195** |
| H7 | .493** | .440** | .277** | .430** | .334** | .402** | .363** | .294** | .335** |
| H8 | .301** | .306** | .203** | .309** | .225** | .291** | .338** | .265** | .205** |
| H9 | .255** | .279** | .190** | .242** | .197** | .262** | .267** | .257** | .248** |
| H10 | .330** | .393** | .281** | .345** | .229** | .393** | .303** | .307** | .263** |
| H12 | .332** | .342** | .316** | .323** | .493** | .292** | .266** | .260** | .274** |
| H13 | .415** | .412** | .372** | .474** | .325** | .365** | .351** | .323** | .315** |
| H11 | .345** | .371** | .355** | .388** | .309** | .368** | .341** | .369** | .307** |
| H14 | .174** | .193** | .148** | .176** | .157** | .202** | .141** | .159** | .157** |
| H15 | .145** | .162** | .149** | .184** | .131** | .181** | .172** | .192** | .100** |
| H16 | .173** | .199** | .195** | .179** | .252** | .172** | .147** | .170** | .187** |
| H17 | -0.043 | -0.023 | 0.002 | -0.030 | -0.014 | -0.015 | -0.027 | -0.009 | -0.044 |

**p<0.001
